# The Role of Climate Change Adaptation in Enhancing Household Food Security: A Case Study of the Hamassa Watershed Agroecologies, Southern Ethiopia

**DOI:** 10.12688/f1000research.160204.1

**Published:** 2025-02-11

**Authors:** Tegegn Bergene, Belay Simane, Meskerem Abi

**Affiliations:** 1 Center for Foos Security Studies, Addis Ababa University College of Development Studies, Addis Ababa, Addis Ababa, 1176, Ethiopia; 2 Department of geography and Environment Studies, College of Social Science and Humanities, Woalita Sodo University, Wolaita_sodo, South Region, 1138, Ethiopia; 3 Center for Environment and Development, Addis Ababa University College of Development Studies, Addis Ababa, Addis Ababa, 1176, Ethiopia

**Keywords:** Climate change adaptation, Households food security, Smallholders farming, Agroecoloogical zones, Hamassa watershed, Southern ethiopia

## Abstract

**Background:**

Climate change adaptation is an incomparable prior measure to tackle unpreventable climate calamities to enhance smallholder farming and food security. This empirical study assesses smallholder farmers’ adaptation options to climate change or variability for achieving food security.

**Methods:**

Data were gathered from a survey of 328 respondents, selected randomly and proportionally from three different agro-ecological zones. Additional qualitative insights were collected through focus group discussions and interviews with key informants to reinforce the findings. The multinomial endogenous switching regression, independent t-test and the instrumental variable (2sls) regression were used as method of analysis

**Results:**

The result indicated that ACAC impacted food security positively and significantly in the study area at a percent rate of 12.4, 16.3,18 and 27.7 when households adopting one, two, three, and four ACAC, respectively, in the HFBM case, and the same meaning was obtained from other food security measuring tools. However, the rate and manner of change differ at different agroecologies, signifying careful discernment when applying ACAC at different spacial areas, especially in agroecology. The change in agroecology declares that midlands have a negative likelihood propensity for climate adaptation compared to highlands, while lowlands have positive and insignificant implications. The mean comparison from the independent t-test showed statistically significant adopters and non-adopters food security measures, which also informed the positive contribution of CACA on households’ food security. Interestingly, factors such as distance to water sources, land size, ox ownership, crop income, and access to credit influenced food security in diverse ways depending on regional and contextual specifics.

**Conclusions:**

Thus, ACAC impacts food security differently at different rates in different agroecologies in the area. Integrated and tailored technical, institutional, and policy interventions are needed to tackle the calamities of climate change leap to smallholder farming and food security

## 1. Introduction

Climate change presents paramount negative stress to global food security; smallholder farmers are among those who face climate distress, depend on rain-fed agriculture, and produce limited adaptive capacity. Low agricultural productivity and food availability shortage are caused by the upscaling frequency and intensity of extreme weather events, coupled with shifting climatic patterns, which exacerbate the vulnerability of these smallholder farmers ( Ogundeji, 2022). Smallholder/small-scale farming is considered a cornerstone for agricultural production, environmental sustainability, and food security in Africa ( Nkansah-Dwamena, 2024; WHO, 2022). Though the continent Africa contributes less to anthropogenic climate change, it receives the highest/damaging stress in the agriculture sector, in turn, food security, which is the backbone of the economy ( Ayugi et al., 2022; Teklu et al., 2024). As a result, sub-Saharan Africa is in a vicious cycle of food insecurity, a problem that has become the ongoing central agenda of agricultural development.

Ethiopia’s smallholder agricultural production and food security are seriously threatened due to climate change ( Belay et al., 2021; Gemeda et al., 2021). Drought and other climate extremes have affected the country frequently ( Esayas et al., 2019; Zeray & Demie, 2016), though its population has increased by 49% within the last 20 years. In the context of high population growth in the country, having severe climate stress on unmodernized rain-fed smallholder farming proclaims a dark future unless checked with integrated hands and civilized techniques.

Many climate adaptation strategies are being implemented in response to climate change, like climate resilience crops, water management, and crop diversification to foster resilience among smallholder farming ( Abraham et al., 2014; Makate et al., 2019; Mujeyi et al., 2021). In smallholder farming, increased agricultural productivity means ensuring food security in return and building capacity for the next production steps ( Mujeyi et al., 2021). Smallholders’ decision to adopt climate change adaptation strategies in rural contexts fully intends the benefit of achieving food security ( Mujeyi et al., 2021). Thus, the adoption of climate change adaptation strategies in the lives and livelihoods of smallholder farming is highly linked to increased crop production, livestock production, and other non-food items production like fuel wood and grasses for fodder, which are sponsoring income from their land possession for food purchase during food shortage.

Traditional smallholders’ agricultural practices, respective of spatial variation, paved the way for easy and quick accommodation of the recent informed decision on the practices of adopting climate change adaptation strategies ( Altieri & Nicholls, 2017). The careful consideration of spatial variation in smallholder rural farming settings is crucial to tackling the climate change calamities that leap to agricultural production and food security, which has been confirmed by empirical studies ( Alemu et al., 2017; Cai et al., 2020). In the spatial variation realm, the issue of agroecology as a factor is more sound when dealing with smallholder farming and climate change ( Adane et al., 2015). Agroecology, with its well-functioning ecosystem processes and services by improving agroecosystems through harnessing natural processes ( Gallardo-López et al., 2018), is among the first stand solutions to smallholder rain-fed destitute Ethiopia farming ( Belete et al., 2023) by lowering the cost of production, increasing productivity and approaching cordially to food security ( Alemu et al., 2017).

Most existing studies have focused on the overall impact of climate change on agricultural productivity or specific adaptation practices in isolation ( Adane et al., 2015; Adenle et al., 2015; Thornton et al., 2018). The differential impact of adaptation measures across varying agroecological contexts and their influence on household food security has received relatively less attention. However, the effectiveness of these strategies in safeguarding household food security across diverse agroecological zones remains underexplored. Thus, connecting agroecology, climate change adaptation, and food security with its standard measure in smallholder rain-fed farming is a new and fascinating approach, adding experience and knowledge to the lack of literature in the area.

This study aims to fill this gap by systematically analyzing the impact of climate adaptation strategies on household food security among smallholder farmers across different agroecological zones. By focusing on various agroecologies, the study seeks to capture the variability in climate impacts and the differential effectiveness of adaptation measures. Furthermore, the research considers the complex interactions between socioeconomic factors, such as access to resources and markets, and the adoption of adaptation strategies.

The findings from this study will provide valuable insights into the most effective adaptation strategies for enhancing food security among smallholder farmers in varying agroecological contexts. Additionally, the research will contribute to the broader discourse on climate adaptation by highlighting the importance of context-specific approaches that consider the unique challenges and opportunities within different agroecological zones. By addressing these critical issues, the study aims to inform policy and practice, guiding the development of targeted interventions that can strengthen the resilience of smallholder farming systems and contribute to global food security in the face of climate change.

## 2. Methods

### 2.1 The study area description

2.1.1 Location and physical characteristics

**Figure 1.**
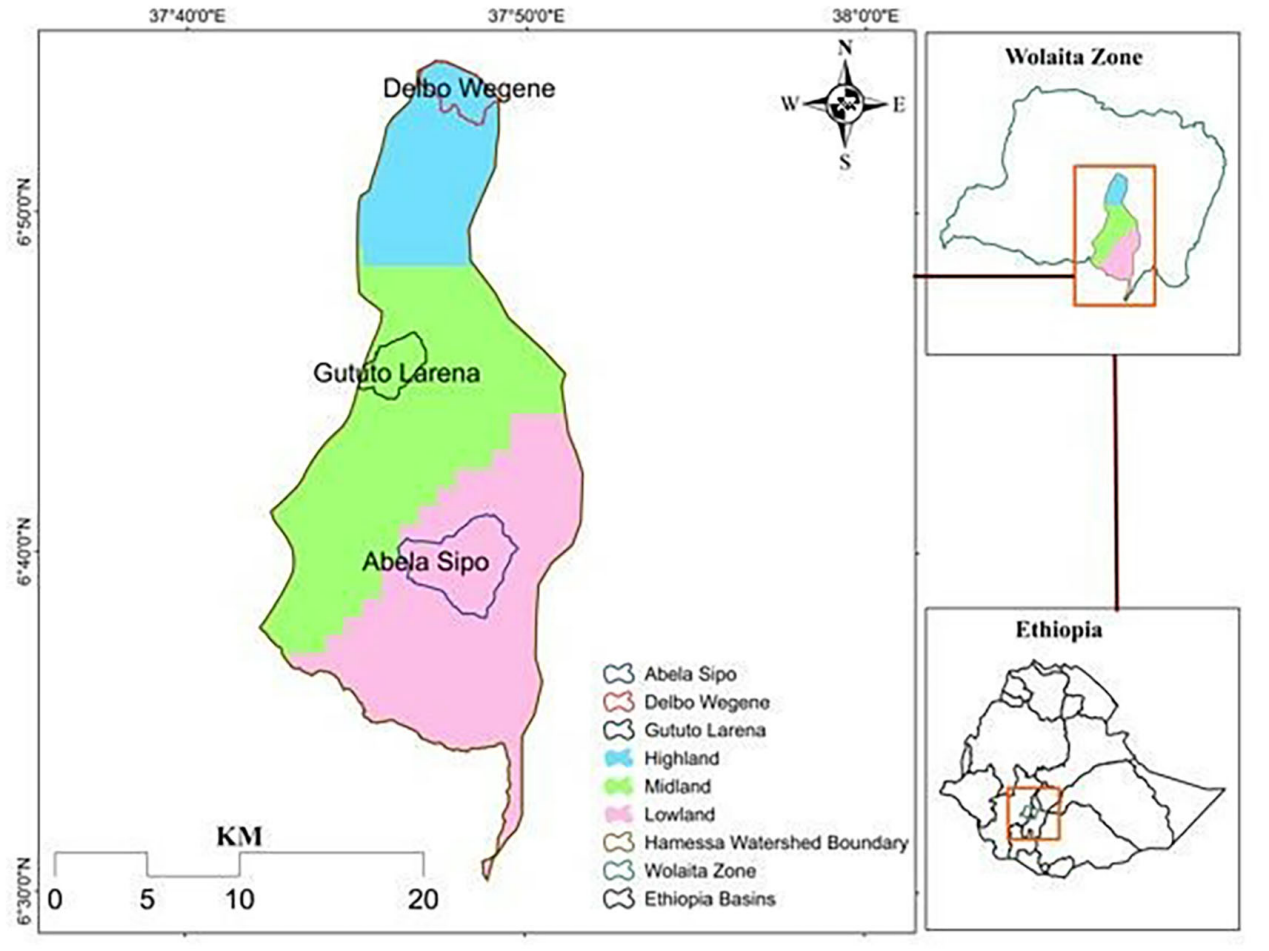
Location map of the study area.

This study was conducted in the Hamassa watershed, which lies within 60 31′13″ to 60 54′28″ N of Latitude and 370 42′01″to 370 53′23″E of longitude. The watershed is located in the Wolaitta Zone, starting from central Wolaitta to the southeast, and shares areas from districts of Sodo Zuriya, Humbo, Abala Abaya, Hobicha (a small section is included), and some part of Sodo town administration (see Figure 1). It is 330 km from Addis Ababa, the capital city of Ethiopia. The watershed’s elevation ranges from 2850 meters average mean sea level (used as mamsl; hereafter) on top of Damota mountain to 1000 mamsl around Lake Abaya. The upper stream parts of the watershed are characterized by mountain and dissected terrain with steep slopes, and the lower stream part that includes the midland and lowland agroecology has gentle slopes with decreasing elevation trend till Abaya Lake. The higher altitude in the watershed is to the northern part, and the elevation continuously decreases to the south-eastern part till the south tip of the watershed. Based on traditional classification, it can be grouped into three agroecological zones (AEZ): highland, midland, and lowland ( Hurni, 1998). The midland and lowland agroecology are dominant in the watershed. The watershed total area is 375.75 km ^2^. The area shares in sq. km of highland, midland, and lowland agroecology are 61.97, 158.60, and 155.17. Soil types in the study area are Fluvisols, Leptosols, Luvisols, Nitisols, and Verisols. The broader area of the watershed is covered with vertisols, which have 325.62 km ^2^ of the total 375.75 km ^2^.

2.1.2 Farming activities

The farming system of the Hamassa watershed is a subsistence-level rain-fed mixed crop-livestock production system. The Hamassa watershed’s two crop-growing rainy seasons mirror Wolaita’s microsplit characteristics. ‘Kiremt’ is the major rain season, which is equally important in crop production, but crops are less produced than in the ‘Belg’ season due to its cold temperature and intensive rain. The ‘kiremt’ rain includes June, July, and August, whereas February to May is the ‘Belg’ rain season, a major production season, but rain is less than ‘kiremt’. The mean annual rainfall varies from 801 mm at Bilate tena to 1400 mm at Wolaitta Sodo. The long-term annual average rainfall and maximum and minimum temperature are 1276 mm, 260C, and 140C, respectively. There is no significant temperature variation across the Zone, but the rainfall variability is very high, and its amount is continuously decreasing to the east till the Bilate area; contrary to this, the amount of rainfall is high around Damota mountain due to the Orographic effect ( Brandt et al., 2012; Yesuph et al., 2023). The precipitation pattern in the studied area is entirely influenced by the equatorial climate systems of the Indian and Atlantic oceans ( Brandt et al., 2012).

2.1.3 Population pressure

Brandt et al. (2012) explained that population pressure, intense farming, and high erosion seriously affect the area, especially the upper part of the mountain. Due to this pressure, natural vegetation, wild bovids, carnivores, and other huge creatures can only be seen in or close to ravines, rocky outcrops, steep slopes, and other locations that are too difficult to settle, plow or hoe. The population of Wolaita is estimated to be 2,353,427, and the crude population density of the Zone is 450 per square km. The most densely populated woredas are Damot Pulasa (722/km ^2^) ( Dana et al., 2020) and Humbo (194/km ^2^) ( Regasa, 2015). According to the 2006 and 2007 estimations, Wolaitta Zone population density is 450 and 464, respectively ( Dana et al., 2020).

### 2.2 Ethical approval statement

The study was granted ethical clearance by the College of Development Studies Institutional Review Board (CoDS-IRB) at Addis Ababa University, and was found to meet the necessary standards, thereby qualifying for Ethical Clearance with No. 035/01/2023 date November 08, 2023. The ethical clearances and standards of the CoDS-IRB mandate that informed consent be obtained from participants, and permit researchers to implement appropriate informed consent procedures, including oral, verbal, or written. Thus, with the IRB’s approval, oral consent was obtained from the participants for this study. The participants’ oral consent was obtained through an audio recording device, specifically the ZTE Blade A71 mobile device (model-ZTE A7030 with Serial number 320225317551). The decision to forego ethical clearance was deemed irrelevant, as adherence to ethical standards and considerations, especially in the context of publication processes, is crucial. Before data collection, the study objectives were clearly communicated to the respondents, ensuring their identities remained confidential in all documentation. Oral consent was obtained from those who volunteered and trusted the project’s ethical declaration following the IRB’s consent, which through informed consent suggested oral consent that I have taken an audio record, as mentioned in the preceding sentences. Oral consent was sought due to some respondents’ inability to read and write and to address time constraints in reaching all respondents

### 2.3. Sampling procedure and Data sources

A multi-stage sampling procedure was applied to select representative samples. The watershed understudy has been chosen using purposive sampling, and the three sample kebeles were randomly selected from three AEZs within the watershed (each Kebele representing the three different agroecology). Finally, the representative sample households were randomly selected proportionately to total household heads and the gender of household heads. The sample size was determined using the ( Cochran, 1977) formula to calculate a representative sample for proportions as:

$$n_{0}=\frac{Z^{²}\mathrm{pq}}{e^{²}}=\frac{{\left(1.96\right)}^{2}\left(0.5\right)\left(0.5\right)\;}{{\left(0.5\right)}^{2}}=384.16$$

Where, *n*_0_ = the sample size; *z* = the selected critical value of desired confidence level; *p* = the estimated proportion of an attribute that is present in the population; *q* = 1− *p* and *e* = the desired level of precision.

Based on ( Cochran, 1977) sample size determination techniques, 328 sample households were selected for the study.

$$n=\frac{n_{0}}{1+\frac{n_{0}-1}{N}}=\frac{384.16}{1+\frac{384.16-1}{2244}}=328$$

### 2.4 Data collection

Quantitative and qualitative data were collected from primary and secondary sources to support the research project, respectively. The quantitative data (primary data) for this research was mainly generated from the structured household survey, and the qualitative data were collected from key informants’ interviews (KII, hereafter), Focus Group Discussions (FGD, hereafter), and observation. The qualitative data as secondary sources were gathered from different literature and reports from government offices.

### 2.5 Food security standard proxy measurement

There are many food security proxy measurements to address the complex and multidimensional food security; various proxy measures are formulated for each component of food security, i.e., food availability, access, utilization, and stability. Among them, we used in this article the Household Food balance model measured in kilocalories (FBM), Coping strategy, and household Diet Diversity score (HDDS), each of them representing food availability, food access, and food utilization respectively ( Dianjaya & Mukti, 2022; Jones et al., 2013; Leroy et al., 2015; Mulugeta, 2010).

HFBM is a mathematical equation used to compute the net food available (NFA), which can be determined with three steps: First, HFBM determines NFA for each household; it is the difference between the gross available food and food disposed of for various reasons. Second, the NFA was converted to total kilocalories for each household and then to Adult Equivalent (ADE) based on conversion factors provided by Ethiopian Health and Nutrition Research Institute food composition table (EHNRI) FDRE 1996 document (as cited in Adane et al., 2015, p. 362). Third, the kilocalories per kilogram calculated in step two compared with the minimum per day per ADE subsistence calories required by an adult to live a healthy and active life in Ethiopia.

A modified Household Dietary Diversity Score (HDDS) was computed for a household based on their household food consumption of various food items/twelve food groups during the preceding 7-day period. Thus, any item consumed by a household within the previous week contributed to the household’s score. The modified HDDS is a continuous score ranging from 0 to 12, representing the household’s diversity of food. Each food group is assigned a score of 1 (if consumed) or 0 (if not consumed). The household score will range from 0-12 and is equal to the total sum of food groups consumed by the household. Food groups considered in the calculation were cereals, roots and tubers, any vegetables, any fruits, any beef, any egg, fish or shellfish, food from (beans, lentils, pea or nuts), milk and milk products, food made of (oil, butter or fat), any sugar or honey, and condiments (coffee and tea).

The coping Strategy Index (CSI) is a behavioural food security measurement type. It arises from the fact that there are several common behavioural responses to food insecurity by households at the time of food shortages. These responses are called the coping strategy. This method has 13 standard questions designed in CSI, recognised as standard coping behaviours regardless of geographical location ( Maxwell & Caldwell, 2008). These questions are broadly categorised into four: 1) dietary change, 2) increase in short-term food availability, 3) decrease in the number of people in the household and 4) rationing strategies. Out of 13 questions, this study selected relevant behavioural questions specific to the study area. Each survey household is asked whether they have used these coping strategies during the last 30 days, and a follow-up question of how often out of the 30 days. The frequency/severity value ranges from 0 to 4, multiplied by the weight given to each item to the score at each household level.

### 2.6 Conceptual framework

The multinomial endogenous switching regression (MESR) is applied in this empirical study for it accounts for choices of adopting adaptation strategies to climate change is possibly endogenous, which means unobserved factors affecting the choice of adopting climate change adaptation strategies may also affect the outcome variable, which is food security. This model can manage the heterogeneity of choices in the adoption of climate change adaptation strategies and their influence on food security. Therefore, the conceptual framework of this study lays its foundation on the household’s decision-making of adopting climate change adaptation strategies with risk and uncertainty in the context of climate change, which is directly based on the economics theory of utility ( Stigler, 1950). The decision of households to adopt the particular adaptation choice is influenced by the households’ characteristics, economic factors and environmental factors ( Tesfaye & Seifu, 2016). What makes these decision complex is that the tread-off between the perceived benefit/utility and cost in adopting adaptation among the given choices. The choice of adoption is directly related to food security, for example, any strategy enhancing soil fertility improves crop production, increasing food security ( Tesfaye & Seifu, 2016), and conversely, any adopting adaptation choices that is resource-intensive may divert resources from food production, potentially leading to food insecurity ( Ires, 2021). To estimate its impact we followed the two step model: firstly, the multinomial logit selection model for different choices of adopting climate change adaptation strategies, and secondly, we tried to estimate the average treatment effect of adopting ordered adaptation strategies (adopting one strategy or two strategy or three strategy or four strategy) on food security. Moreover, a 2sls regression is applied to examine the endogenous variable and exogenous variable effect on outcome including instrumental variable to correct errors that can arise from endogeneity. The mean difference between climate change adaptation strategies adopters and non-adopters examined using independent t-test.

### 2.7 Analytical framework

Smallholders were asked what adaptation strategies they use to reduce the impact of climate change on production and food security in their framing with chance of multiple response, and they responded five main adaptation strategies used widely and frequently in the area: which are crop diversification, using fertilizer, improved seed varieties and soil and water conservation and shifting crop calendar. Based on their replies household were identified to those groups who has used no adoption, one adoption or two or three or four adaptation strategy. This techniques are used by empirical study by grouping treatment variable (to examine what would the more treatment do on outcome variable) ( Hailu et al., 2021) to estimate the effect of no adoption, one adoption or two or three or four on outcome variable. Besides, the researchers close knower of the areas and the pilot study, the consultation of district level rural development offices and the ample sources of literature review on climate change adaptation strategies in rural smallholders setting ( Aryal et al., 2020; Belay et al., 2017; Gebre, Amekawa, & Fikadu, 2023a; Gebre, Amekawa, Fikadu, et al., 2023b; Nigussie et al., 2018; Ojo et al., 2022; Tesfaye, 2019a) comprehensively assessed to differentiate which adaptation choices to be considered in the area under this research project. The exhaustively identified adaptation strategies were contextualized to the study area as presented above and commonly used five main adaptation strategies selected for this empirical studies, its structure as presented in connection to food security guided to have the model analysis in this paper.

**2.7.1 Multinomial logit selection model**

Smallholders choose among the ACAC alternatives that was expected to have a return of highest utility. They adopt among the given ACAC alternatives by comparing the utility *U*_*i*_ provided by m alternatives choices. Thus, households’ utility is indirectly observed through ACAC choice, which increase their food security situation (this utility is a latent). Among given alternative *m*, the household *i* demand to choose alternative strategy *j* is specified as $U_{\mathrm{ij}}>U_{\mathrm{im}}m\neq j,\text{or equivalently}\;\Delta U_{\mathrm{im}}=U_{\mathrm{ij}}-U_{\mathrm{im}}>0m\neq j.$ The expected *U*_ij_* is a latent variable derived from the adoption of ACAC “ *j*”, which can be determined by observed households’ characteristics, economic factors, environmental factors and also unobserved characteristics( $\varepsilon_{\mathrm{ij}}$ ) ( Bourguignon et al., 2002)

$$U_{\mathrm{ij}}^{*}=X_{i}\beta_{j}+\varepsilon_{\mathrm{ij}}$$

Where *U*_*ij*_ is perceived utility of household derived from ACAC adoption, *X*_*i*_ is vector affecting perceived utility, $\beta_{j}$ is a parameter, and $\varepsilon_{\mathrm{ij}}$ are residuals.

If *U*_ij_* is the highest, the ACAC *j* is chosen for farming household *i*. Therefore, the family select a different ACAC *j* instead of implementing any other ACAC option *m* if ( Bourguignon et al., 2002)

$$\begin{array}{c} U=\left\{ \begin{array}{cc} 1 & \;\text{if}\;U_{i1}^{*}>\max_{m\neq j}\;\left(U_{\mathrm{im}}^{*}\right)\;\text{or}\;\eta_{i1}<0\\ \vdots & \vdots \\ j & \;\text{if}\;U_{\mathrm{ij}}^{*}>\max_{m\neq j}\;\left(U_{\mathrm{im}}^{*}\right)\;\text{or}\;\eta_{\mathrm{ij}}<0 \end{array}\;\text{for}\;\mathrm{all}\;m\neq j\right. \end{array}$$

where $\eta_{\mathrm{ij}}\neq \max_{m\neq j}\;\left(U_{\mathrm{im}}^{*}-U_{\mathrm{ij}}^{*}\right)<0$ . It implies that the ith farmer will utilize package j to maximize expected benefit if it has a higher expected utility than any other package *m* ≠ *j*.

A multinomial logit model can be used to specify the likelihood that farmer *i* with attributes *X* will select ACAC *j*, following ( Kassie et al., 2015)

$$P_{\mathrm{ij}}=\mathrm{pr}\left(\eta_{\mathrm{ij}}<0|X_{i}\right)=\frac{\exp\left(X_{i}B_{i}\right)}{\sum_{m=1}^{j}\;\exp\left(X_{i}B_{i}\right)}$$

**2.7.2. Endogenous switching regression**

Being this model a grand frame, a two-stage separate equation has been applied for endogenous switching modeling ( Gorst et al., 2018): firstly, the selection model for ACAC with different options having binary variables. The latent variable ( m*)(specified as follows ( Kassie et al., 2015), which gains expected utility from adopting ACAC with respect to not adopting:

$$M^{*}=Z_{i}\alpha +\varepsilon_{i}\;\text{with}\;M_{i}=\left\{ \begin{array}{c} 1\;\;\text{if}\;M_{i}>0\\ 0\;\;\text{Otherwise}\; \end{array}\right.,$$

Where *M* ^*^ equals 1 with its binary indicator if a household adopt alternative ACAC and zero otherwise; *Z* is a vector of factors influencing ACAC; *α* is a vector of parameters to be estimated; and *ε* is the error term.

Secondly, the outcome estimation equation that run separate regime for ACAC and not to adopt any by splitting the endogenous model, doing so its equation has done as specified below:

$$\left\{ \begin{array}{cc} \;\text{Regime}\;1:Q_{i1}=Z_{i}\alpha_{1}+U_{i1} & \;\text{if}\;I=1\\ \vdots & \\ \;\text{Regime}\;j:Q_{\mathrm{ij}}=Z_{i}\alpha_{j}+U_{\mathrm{ij}} & \;\text{if}\;I=j \end{array}\right.,$$

Where ( *Q*’ _1_) s are the outcome variables of the ith farmers in regime *j*, and the errors ( *u*’s) are distributed with $E\left(U_{\mathrm{ij}}|X,Z\right)=0\;\text{and}\;\mathrm{var}\left(U_{\mathrm{ij}}|X,Z\right)=\delta_{i}^{2}\cdot Q_{\mathrm{ij}}$ is observed if and only if technology package *j* is adopted, which occurs when $U_{i1}^{*}>\max_{m\neq j}\;\left(U_{\mathrm{im}}^{*}\right)$

**2.7.3. Average treatment effect by using counterfactual**

In the endogenous switching regression, alternative ACAC are considered as treatment group with their counterfactual estimation to estimate the ACAC impact on food security. In this context the treatment effect estimation get due attention with average treatment effect (ATE) and average treatment effect for treated (ATT). Having this in mind, we went through two counterfactual estimation approach. First: how would FBM, HDDS and CS change if everyone in the sample treated with particular ACAC relative to non-adopters is measured to estimate ATE. Second: ATE is also seen in this empirical study by answering the question of how would FBM, HDDS and CS change if everyone in the sample treated with particular ACAC relative to if he would adopt the next higher ACAC level. Both ATE approach/counterfactual estimation are used to have in-depth insights of the treatment effect in the study area.

1.
$E\left(\frac{Q_{\mathrm{ij}}}{I}=j\right)=Z_{\mathrm{ij}}\alpha_{j}+\sigma_{j}\lambda_{\mathrm{ij}}$ : Adopters with adoption (actual),
2.
$E\left(\frac{Q_{i1}}{I}=1\right)=Z_{\mathrm{ij}}\alpha_{1}+\sigma_{1}\lambda_{i1}$ : Non-adopters without adoption (actual),
3.
$E\left(\frac{Q_{i1}}{I}=j\right)=Z_{\mathrm{ij}}\alpha_{1}+\sigma_{1}\lambda_{\mathrm{ij}}$ : Adopters had they decided not to adopt (counterfactual),
4.
$E\left(\frac{Q_{\mathrm{ij}}}{I}=j\right)=Z_{\mathrm{ij}}\alpha_{j}+\sigma_{j}\lambda_{i1}$ : Non-adopters had they decided to adopt (counterfactual),
5.
$\left(\frac{Q_{\mathrm{ij}}}{I}=j\right)=Z_{\mathrm{ij}}\alpha_{j+1}+\sigma_{j+1}\lambda_{\mathrm{ij}}$ : Adopters had they decided to adopt the next higher level ACAC

Equation one and two under this section is estimating actually observed expected outcome of adopters and non-adopters, while, equation three to five are estimating expected outcome of counterfactual.

## 3. Results

Among the identified independent variables, the development agents’ support, climate change perception, education level of household head, and age of the household heads were found to be affecting both the treatment variable (adoption of climate change adaptation strategies) and outcome variable (food security). Thus, they were used as instrumental variables affecting the choices adopting climate change adaptation strategies, affecting food security through adopting adaptation. As indicated in Table 1 below, a unit increase in adopting climate change adaptation strategies increases the kcal of households by about 1354.73 on average at 0.000/most significant level, which signifies the noticeable role of climate change adaptation on households’ food security. In the same way, an increase in the number ox and migration to shortage of food increases the probability of household food insecurity at the most significant and significant level, while, an increase in market distance, female-headed households, poor household health and shortage of water availability decrease the probability of households food security at most significant, highly significant and the last two are at significant level respectively.

**Table 1.** Gross ACAC effect on households food security using 2sls.

Instrumental variables (2SLS) regression	Number of obs = 328
	Wald chi ^2^(21) = 470.58
	Prob > chi ^2^ = 0.0000
	R-squared = 0.5757
	Root MSE = 1010.9

Foodsec kcal	Coef.	sign
Adopting CC adaptation level	1354.73	0.000 ^***^
HHS size	-5.68	0.839
Dependency ratio	-59.45	0.398
Crop income	-0.0066	0.559
Livestock income	0.018	0.092 ^*^
Off/on farm income	.0059	0.452
Num of ox	385.68	0.000 ^***^
TLU	80.97	0.158
Market distance	-39.58	0.009 ^***^
Water distances	-34.84	0.304
Cultivated land size	40.34	0.412
Total land size	11.59	0.541
Agroecology zone		
2	-2.44	0.990
3	270.70	0.153
hhs sex	-375.03	0.048 ^**^
Migration status	239.55	0.059 ^*^
Credit access	6.04	0.960
HHS health	-189.21	0.033 ^**^
Water availability	-273.10	0.079 ^*^
SWC practices	189.09	0.146
Social capital	-187.64	0.278
-cons	-509.23	0.311
**Instrumented: adaptation level**		

Note: ^*, **,^ and ^***^ are statistically significant, highly significant, and most significant, respectively.

In all agroecologies, all proxy measures of food security (three), their t-value (mean difference) food security is significant between adopters of climate change adaptation strategies and non-adopters among households in the study area at the most significant level both the households FBM measure and HDDS (See Table 2). The negative t-value of the coping CS (proxy measure) tells us the cumulative coping strategy index for non-adopter households is by far higher than the adopters because they need to cope with the existing harsh food shortage with tread off their fixed asset or other livelihood assets, and, which sometimes may cause irreversible risk, but their mean difference is statistically not significant as shown in Table 2.

**Table 2.** Independent t-test of climate change adaptation adopters and non-adopters food security measure of households.

Variable (food security proxy measures)	mean	std	t-value	significance
**Highlands**				
Kcal	1965.7	476.9	10.115	0.000***
HDDS	4.8	2.2	2.149	0.034***
CS	-10.3	7.6	-1.3	0.180ns
**Midlands**				
Kcal	938.5	140.4	6.7	0.000***
HDDS	1.8	0.42	4.2	0.000***
CS	-9.1	7.1	-1.3	0.203ns
**Lowlands**				
Kcal	1574.8	158.9	9.9	0.000***
HDDS	1.8	0.55	3.4	0.002***
CS	-8.3	6.5	-1.3	0.206ns

Note: ns and ^***^ are statistically not significant and most significant, respectively.

In the multinomial logistic model (logit) in Table 3, the Critical Value from the Chi-Squared Distribution is 60.4, while LRX2 of logit below is (223.64) which by far exceeds the critical value from the χ ^2^ distribution shows the model with predictors fits significantly better than the null hypothesis with p-value 0.000. Moreover, the higher difference between the null model value -497.12306 and the full model -385.3008 indicates that the model explains the data better.

**Table 3.** The multinomial logistic regression.

Multinomial logistic regression	Number of obs = 328
	LR chi ^2^(80) = 223.64
	Prob > chi ^2^ = 0.0000
	Log likelihood = -385.30086 Pseudo R ^2^ = 0.4922

Adopting CC adaptation level	Coef.	Std.err.	sign
0 base category			
1			
hh size	-.116	.08	0.197
Dependency ration	-.264	.221	0.233
Crop income	-.0002	.0001	0.120
Livestock income	.0001	.00006	0.086*
Off/nonfarm income	.0002	.00002	0.335
Ox	-17	.36	0.646
TLU	.301	.17	0.092*
Market distance	-.13	.058	0.834
Water distance	.33	.18	0.068*
Cultivated land size	.28	.31	0.375
Total land size	-.59	.053	0.271
Agroecology zone			
2	-.302	.63	0.636
3	1.15	.74	0.124
sex respondent	1.44	.71	0.043***
Migration	-.292	.45	0.520
Credit	.260	.43	0.552
hh health	.634	.37	0.089*
Water availability	.865	.45	0.060*
Social capital	1.083	.61	0.081*
cons	-2.270	1.400	0.102
**2**			
hh size	-.19	.095	0.049**
Dependency ration	-.302	.250	0.228
Crop income	-.0006	.00004	0.001***
Livestock income	.0009	.00005	0.052*
Off/nonfarm income	-.0008	.00002	0.561
Ox	-.0598	.38	0.877
TLU	.504	.18	0.005***
Market distance	-.083	.06	0.179
Water distance	.462	..18	0.013***
Cultivated land size	.443	.31	0.163
Total land size	-.095	.067	0.159
Agroecology zone			
2	-.24	.65	0.719
3	.74	.77	0.343
sex respondent	.804	.67	0.234
Migration	-.895	.48	0.063*
Credit	-.3997	.45	0.384
hh health	.325	.37	0.389
Water availability	1.23	.49	0.012**
Social capital	.2808	.62	0.653
cons	.296	1.406	0.834
**3**			
hh size	-.408	.121	0.001***
Dependency ration	-1.68	.516	0.001***
Crop income	-.0008	.00004	0.022**
Livestock income	.0006	.00006	0.087*
Off/nonfarm income	.0009	.00003	0.041**
Ox	.347	.427	0.418
TLU	.843	.206	0.000***
Market distance	-.058	.068	0.403
Water distance	.019	.252	0.943
Cultivated land size	.588	.325	0.074*
Total land size	-.109	.097	0.261
Agroecology zone			
2	-1.363	.804	0.090*
3	.445	.881	0.614
sex respondent	1.378	.889	0.123
Migration	-.686	.572	0.231
Credit	-.524	.529	0.322
hh health	.329	.415	0.429
Water availability	1.767	.622	0.005***
Social capital	.523	.780	0.504
cons	.637	1.699	0.708
**4**			
hh size	-.371	.147	0.012**
Dependency ration	-.412	.454	0.364
Crop income	-.0009	.00005	0.001***
Livestock income	.0003	.00007	0.059*
Off/nonfarm income	.0007	.00004	0.080*
Ox	.280	.473	0.553
TLU	1.266	.243	0.000***
Market distance	-.006	..078	0.930
Water distance	.125	.279	0.652
Cultivated land size	.464	.371	0.211
Total land size	-.089	.116	0.445
Agroecology zone			
2	-2.157	1.072	0.045**
3	1.646	1.160	0.156
sex respondent	-.183	.916	0.841
Migration	.554	.673	0.411
Credit	-.008	.635	0.990
hh health	.553	.469	0.239
Water availability	3.17	1.206	0.008**
Social capital	.348	.952	0.714
cons	-3.036	2.162	0.160

Note: ^*, **,^ and ^***^ are statistically significant, highly significant, and most significant, respectively

Regarding the interpretations of the independent variables in the model, variables like hh size, dependency ratio, crop income, ox, market distance, total land size, sex, migration, and soil and water conservation (swc) show the decreased likelihood of adopting climate change adaptation strategies of choice category one when their value increase compared to the baseline category (o adoption). But, for others like livestock income, TLU, sex, hh health, water availability, and social capital, a unit increase in them results in an increased likelihood of adopting choice one when compared to adopting 0. Switching male to female means increasing the likelihood of adopting choice one is more significant than others. This might be that in the study area neighbors and relatives provide a little support and appraisal for widowed (female headed household heads) via ploughing and other similar agricultural activities, which is at ACAC one level but not in others indicate widowed receiving a little aid for she is needy. This situation expressed in the key informant’s interview from midland:

*We used to support both our asset and labour for needy people in the area, but now, situations changed and the aid being given to needy ones significantly decreasing because smallholders stressed to their own challenges of farming and food security*

In adopting choice two, categories (crop income, TLU, and water distance) and (hh size and water availability) and (livestock income and migration) are affecting adopting choice two at the most significant, highly significant, and significant levels, respectively. Among them, TLU and water availability contribute positively to the likelihood of adopting choice two when compared to the base category at the most significant and highly significant level,

Adopting climate change adaptation strategies, choice three is likely to decrease and increase (hh size and dependency ratio) and (TLU and water availability) at the most significant level. When it comes to choice four, TLU and crop income affect positively and negatively at the most significant level.

Table 4 below tells the percentage difference in each food security proxy measure in each household, switching from each lower adoption choice level to the next higher level of counterfactual to examine the impact difference on outcome variables. For example, regarding households’ food balance model of kilocalories (FBM-kcal), switching from non-adopting to adopting choice one strategy increases households’ food security by 12.35%. with similar trend when households switch from 1 to 2, 2 to 3, 3 to 4, and 4 to 5, increasing the household’s kilocalories by percent of 16.35, 18.1, 27.72, and -1.87, respectively.

**Table 4.** Percentage difference between factual and counterfactual food security estimation overall study area.

FS proxy measurement	Counterfactual (X)	Factual (Y)	Average adoption effect (Z=X-Y)	Impact(%) A=(Z/Y)*100
**FBM-Kcal **				
0 to 1	6070.049	5399.43	670.619	12.42
1 to 2	5895.748	5067.205	828.543	16.35
2 to 3	5270.61	4463.036	807.574	18.1
3 to 4	5457.528	4273.251	1184.277	27.72
4 to 5	4694.239	4783.371	-89.132	-1.87
**HDDS**				
0 to 1	57.21	53.11	4.1	7.72
1 to 2	42.78	37.12	5.66	15.27
2 to 3	33.29	28.37	4.92	17.34
3 to 4	23..73	16.50	7.23	43.82
4 to 5	11.62	12.16	-0.54	-4.44
**CS**				
0 to 1	39.69	38.52	1.17	3.037
1 to 2	39.27	37.82	1.45	3.834
2 to 3	45.53	44.12	1.41	3.20
3 to 4	36.93	34.86	2.07	5.94
4 to 5	37.95	37.90	0.05	0.13

In the same way, the diet diversity of households increases (better food security status) perpetually when households switch to the next higher level, the same output regarding the coping strategy food security proxy measure, which vividly explains the noticeable positive contribution of adopting climate change adaptation strategies to the food security status of households in the agroecology of the study area.

As presented in Table 6 below, the potential outcome mean (Pomean) explains what the average food security would be if households either adopted or did not adopt the climate change adaptation strategies at each adaptation level. Thus, when compared to non-adapting in each level, adopting climate change adaptation strategies one to four levels is related to an average increase of kcal per individual by 486.55, 911.3, 1960.8, and 3008.8, respectively. The same perpetual increment is seen in the diet diversity indicator of food security at the most significant level. The exact meaning is experienced when the coping strategy index is applied, as seen from the table; the negative sign in this case referring the cumulative coping statutory index of when non-adopting is greater than adopting, inferring households while not adopting means they pay much sacrifice to manage the food shortage sometimes deteriorating livelihood assets irreversibly. Empirical research with similar finding assured the negative relationship between coping strategy and diet diversity score of households ( Saaka et al., 2017).

## 4. Discussion

Through all analysis, be it t-test, instrumental variables (2sls) regression, and the endogenous switching model, the adoption of climate change adaptation strategies positively and significantly contributes to the food security situation in the study area. This finding concomitants with other findings ( Hussein, 2024; Singh, 2024; Tilahun et al., 2023). The perpetual increase of food security likelihoods is observed in the study area when switching to the next higher level of adopting climate change adaptation choices (ACAC), signifying more ACAC means a better food security situation. The potential outcome (POmean) of the food security analysis also indicated the significant role of ACAC in adopting and not adopting climate change adaptation strategies among households in the study area.

More importantly, it is sincerely advisable in this paper to notice the perpetual and proportionate increment of the food security situation of households when adopting the higher level of climate change adaptation choices, which is true in both counterfactual cases either switching each ACAC level to the next higher level or switching each ACAC level to zero. This assentation gets a different meaning at level four, which might be adopting more climate change adaptation strategies if not well-considered capacities, it may cause maladaptation to climate change, affecting food security reversibility.

Further impressive notion as an output from analysis, though ACAC generally contributes positively and significantly to food security in all agroecologies, the trend or manner of change is a bit different that dictates to look into varied agroecologies in different discernment. The more ACAC gives a return of food security at a higher rate and positively in both highlands and lowlands, but its return is positive and very little or slow in midlands. Any additional ACAC of more than 3 results in a decreasing or negative return in the midlands. In contrast, its negative and decreasing return is observed when ACAC is more than 4 in the highlands and lowlands, respectively. This variation might be that the midland’s disproportionate land size and population pressure, including large family size coupled with low capacities, result in low irresponsive and negative food security for more ACAC, which is confirmed by FGDs results indicating midlands have no or minimal natural assets like land and household assets. Moreover, the reason might be also biasness in selecting effective adaptation strategies that correctly set in their social, economic and environmental background, which need to be supported by professionals so as to be effective. The large-sized land in the lowlands ( Belay, 2021) and the intensive land management programs both from government and non-government organizations in the highlands ( Abo & Hailu, 2017; Solomon et al., 2022) may assist the positive high rate food security return for each more ACAC. This finding goes with a study found that midland land agroecology is a more severe food insecure spatial area than the lowlands and highlands ( Bergene, Simane, Abi, et al., 2024b; Giller et al., 2021; Phan et al., 2022)

The independent t-test mean difference between climate change adaptation strategy adopters and non-adopters is statistically most significant in the food security proxy measure of households’ food balance model (household kilocalorie energy) and households’ diet diversity strategy (HDDS), ensuring the climate change strategy adoption significantly contributing to food security in the study area. However, the case is different in the coping strategy proxy measure of food security that the mean difference between climate change adaptation strategy adopters and non-adopters is statistically not significant; this might explain the unique nature of food insecurity of the study area, whose inhabitants, though achieve/fill their food gap with the help of adoption, its success is through the practice of coping strategy. The practice of a coping strategy is mandatory even for better-off groups to achieve their food need in the area. Still, the type and severity of coping strategy vary between adopters and non-adopters, as revealed by the FGD’s explanation that the non-adopters lost their basic assets, including land, to fill the food gap. In contrast, adopters may sell bull, heifers, sheep, and goats for food purchases to fill the gap.

Regarding the determinants of ACAC in the mlogit model, agroecology variation affects the adoption of climate change adaptation strategies; for example, when comparing the midland agroecology to the highlands, it decreases the likelihood of ACAS at any level but levels three and four at 10% and 5% percent significant level, respectively. This finding aligns with other empirical findings that assert the socio-spatial impact on farmers’ choices of climate change adaptation ( Mainardi, 2018; Smart et al., 2023). The lowlands show a propensity of increased likelihood of adoption to climate change when compared to the highlands, but it is statistically insignificant.

The negative coefficient of total land size and income from crop sale against the hypothesis or theory is that smallholder farmers do not sell their crop products unless they are dictated to pay for any debt or to purchase their compulsory non-food items from their market. Farmers in their small plots of land (which seems unfair to sustain the existing large household size) have products at the subsistence level; they have no willful sell of farm products, which was confirmed during the focus group discussion in the study area. Empirical study with similar finding noted that if they sell crops in time of surplus, this exert negative welfare and food access in time of food shortage ( Tesfaye, 2019b). They claimed it with their local saying, “Man who sells crop and animals will not be pitied,” which clarifies how much it is opted not to sell their farm products. However, they find it surplus for the occasion they reserve it for later use knowing that they will face shortage of food in near future, which is even get worse by climate distress. The study area is known for its frequent and severe drought extremities by different authors ( Bergene, Simane, & Abi, 2024a). Thus, they/smallholders do not have income from crop sales for ACAS. The case of total land size negative implication against the expectation would be that the unproductive sections of land like homesteads, lands used for grazing for its dissected and unproductive nature and lands with shrubs included in it, that is why the implication is opposite when using the cultivated land size in the same model (see Table 3), which have positive implication for ACAS. Moreover the negative direction of number ox to Adopting climate change adaptation choices at choice one and two level might be related to poor socioeconomic characteristics of household, where ox or any other livestock if present, that are given by other person for common benefit of milk or cow offspring, and fattening. The scenario is changed when ACAC scaled up to level three and four, whose direction is positive to adaptation. This meaning when ox are their own of smallholders they rear it for farming purpose (ox serves for farming in areas) and its increase/presence increase the likelihood of ACAC; giving positive return of households food security. Empirical studies confirmed that ox-plough has a positive and significant contributor to farming ( Fekede et al., 2016; Melese et al., 2021; Okello et al., 2019), its presence boost and facilitate smallholder farming to adopt climate change adaptation ( Adego & Woldie, 2022).

The positive water distance implication to ACAC might be that the point water source/springs the rural societies use are mainly for home consumption like cooking rather than giving good meaning to ACAC for farming. Market distance can get more attention than water distance in rural setups because inputs for ACAC, like selected seeds and fertilizers (their right time presence has great meaning) ( Destaw & Fenta, 2021; Tessema et al., 2013), and their far distance can affect climate change adaptation negatively, as seen/found out in Table 3.

Therefore, it can be concluded that the adoption of climate change choices has a significant role in sustaining the food security situation of rural smallholder farming as a whole, but differently at different rate in different agroecologies (see Tables 3, 5 and 6), whose role may get great attention than before in the study area since frequent drought and other climate change calamities intense and wide ( Bergene, Simane, & Abi, 2024a; Degefu & Bewket, 2015; Esayas et al., 2019; Malkato et al., 2022; Tessema et al., 2017).

**Table 5.** Treatment effect of ACAC on food security by agroecology.

		Counterfactual (X)	Factual (Y)	Average adoption effect (Z=X-Y)	Impact(%) A=(Z/Y)*100
**FBM-Kcal food security proxy measurement**					
** Highlands**	0 to 1	6462.566	5489.266	973.3	17.7
	1 to 2	5071.656	4067.202	1004.454	24.7
	2 to 3	3480.369	2587.43	892.939	34.5
	3 to 4	4919.858	3765.42	1156.438	30.7
	4 to 5	4303.836	4612.962	-309.127	-6.7
**Midlands**	0 to 1	6825.48	6033.026	792.454	13.13
	1 to 2	5828.864	4972.104	856.742	17.23
	2 to 3	6024.626	5092.818	931.808	18.3
	3 to 4	6396.431	5687.333	709.098	12.47
	4 to 5	3785.499	4325.145	-539.6	-12.5
**Lowlands**	0 to 1	5077.134	4741.413	335.721	7.08
	1 to 2	6330.78	5601.641	729.641	13.02
	2 to 3	6294.66	5501.361	793.299	14.42
	3 to 4	6133.81	4834.561	1299.249	26.87
	4 to 5	5746.352	5198.796	547.556	10.53
**HDDS food security proxy measure**					
**Highlands**	0 to 1	76.11	70.18	5.93	8.45
	1 to 2	51.27	45.14	6.13	13.58
	2 to 3	48.68	42.73	5.95	13.9
	3 to 4	24.56	21.52	3.04	14.1
	4 to 5	12.33	14.14	-1.81	-12.80
**Midlands**	0 to 1	42.74	37.91	4.83	12.7
	1 to 2	21.81	18.58	3.23	17.4
	2 to 3	29.04	24.71	4.33	17.52
	3 to 4	12.84	10.95	5.69	17.3
	4 to 5	-1.02	-.88	-0.14	15.9
**Lowlands**	0 to 1	56.84	54.79	2.05	3.74
	1 to 2	47.53	43.09	4.44	10.30
	2 to 3	20.62	15.78	4.84	30.67
	3 to 4	13.5	10.32	3.18	30.80
	4 to 5	14.28	10.94	3.36	30.71
**CS food security proxy measure**					
**Highlands**	0 to 1	31.21	29.2	2.01	6.9
	1 to 2	37.36	35.8	1.56	4.38
	2 to 3	49.38	47-67	2.01	4.23
	3 to 4	43.91	42.15	1.76	4.18
	4 to 5	33.72	34.25	-0.53	-1.5
**Midlands**	0 to 1	50.47	49.23	1.24	5.51
	1 to 2	36.27	34.64	1.63	4.7
	2 to 3	60.51	59.01	1.5	2.56
	3 to 4	54.75	53.36	1.39	2.6
	4 to 5	45.06	47.23	-2.17	-4.59
**Lowlands**	0 to 1	45.95	43.67	228	5.22
	1 to 2	27.02	25.75	1.27	4.93
	2 to 3	18.53	17.94	0.59	3.29
	3 to 4	50.35	48.97	1.38	2.82
	4 to 5	43.9	42.98	0.92	2.14

**Table 6.** Another conditional treatment effect examines households' CC adoption effect.

Treatment-effects estimation	Number of obs = 328
	Estimator: regression adjustment
	Outcome model: linear
	Treatment model: none

	Robust		
Foodsec kcal	Coef.	Std.err	sign
**ATET**			
**Adaptation level**			
**(1 v 0)**	486.5507	79.24533	0.000***
**(2 v 0)**	911.3229	109.304	0.000***
**(3 v 0)**	1960.833	300.029	0.000***
**(4 v 0)**	3008.841	518.777	0.000***
**POmean**			
**0**	765.6668	61.9684	0.000***
**HDDS**			
**(1 v 0)**	.4815	.2901	0.097*
**(2 v 0)**	.9585	.3383	0.005**
**(3 v 0)**	7.4385	.3484	0.000***
**(4 v 0)**	15.9909	5.5510	0.004**
**POmean**			
**0**	2.2344	.2498	0.000***
**CS**			
**(1 v 0)**	-4.5791	4.595	0.319
**(2 v 0)**	-9.3209	4.7732	0.051*
**(3 v 0)**	-13.0314	4.9577	0.009**
**(4 v 0)**	-14.8217	5.6505	0.009**
**POmean**			
**0**	52.725	5.650	0.000***

Note: ^*, **,^ and ^***^ are statistically significant, highly significant, and most significant, respectively.

## 5. Conclusions and recommendations

The study examined the role of adopting climate change adaptation choices in enhancing food security in Hamassa watershed, southern region, Ethiopia. The study considered 328 sample size randomly and proportionately selected from three agroecological zones in the study watershed. The HHFBM, Diet diversity score and copying strategy were used as food security proxy measure, besides, the multinomial endogenous switching regression, the independent t-test and instrumental variables (2sls) regression were undertakes as methods of analysis to reach out ACAC role in smallholder subsistence farming food security.

Generally, as the data revealed, ACAC impacted food security positively and significantly in the study area at a percent rate of 12.4, 16.3,18 and 27.7 when households adopting one, two, three, and four ACAC, respectively, in all food security measures (HFBM, Diet diversity score and copying strategy) in the study area as a whole. Though positive and significant, the manner and rate of food security change for each level of ACAC vary across AEZs, demonstrating inevitable spatial discernment in the climate change and food security relationship. Another impressive finding in this paper is the socioeconomic, demographic and environmental determinants factors significantly affecting when practicing ACAC. Though their rate vary across each ACAC level, TLU, water availability, sex, livestock income, agroecology and others (see Table 3) determined ACAC in the study area.

ACAC as climate adaptation package can cover a wide spectrum and comprehensive field like soil, income, climate change mitigation, income diversification, and production stability; for farmers to get the maximum benefit from ACAC, smallholders should include all options of climate change adaptation as much as possible with critical scholar guidance/support to reduce malpractice of adaptation.

The independent t-test results testifies the most significant mean difference of food security status between adopters and non-adopters. The integrated NGO and government bodies cooperatively should enhance farmers’ awareness of climate change and climate smart agriculture innovative through different approaches like farmers training center, demonstration site, farmer to farmer learning and experience sharing, climate change adaptation innovation portfolio for each agroecological zones in terms of synergy and trade-off, provide farmers with vegetable and perennial seeds, support to adopt home garden agroforestry, provide them with drought resistance and high yielding crop varieties. As the data indicated in this research paper farmers with increased land size have increased likelihood of food security; hence, land policy need to consider the farm rental land markets, which may help rent-based smallholders to acquire more land for production.

Therefore, it can be concluded that the adoption of climate change choices has a significant role in sustaining the food security situation of rural smallholder farming as a whole, but differently at different rate in different agroecologies (see Tables 3 and 5), whose role may get great attention than before in the study area since frequent drought and other climate change calamities intense and wide ( Bergene, Simane, & Abi, 2024a; Degefu & Bewket, 2015; Esayas et al., 2019; Malkato et al., 2022; Tessema et al., 2017).

## Ethical approval statement

The study was granted ethical clearance by the College of Development Studies Institutional Review Board (CoDS-IRB) at Addis Ababa University, and was found to meet the necessary standards, thereby qualifying for Ethical Clearance with No. 035/01/2023 date November 08, 2023. The ethical clearances and standards of the CoDS-IRB mandate that informed consent be obtained from participants, and permit researchers to implement appropriate informed consent procedures, including oral, verbal, or written. Thus, with the IRB’s approval, oral consent was obtained from the participants for this study. The participants’ oral consent was obtained through an audio recording device, specifically the ZTE Blade A71 mobile device (model-ZTE A7030 with Serial number 320225317551). The decision to forego ethical clearance was deemed irrelevant, as adherence to ethical standards and considerations, especially in the context of publication processes, is crucial. Before data collection, the study objectives were clearly communicated to the respondents, ensuring their identities remained confidential in all documentation. Oral consent was obtained from those who volunteered and trusted the project’s ethical declaration following the IRB’s consent, which through informed consent suggested oral consent that I have taken an audio record, as mentioned in the preceding sentences. Oral consent was sought due to some respondents’ inability to read and write and to address time constraints in reaching all respondents

## Author contribution statement

Authors, Tegegn Bergene, Belay Simane and Meskerem Abi, have contributed to the project from conception and design to final approval.

## Data Availability

Figshare: [‘The Role of Climate Change Adaptation in Enhancing Household Food Security: A Case Study of the Hamassa Watershed Agroecologies, Southern Ethiopia’]. The project contains the following underlying data:
• Climate change and food security (This empirical study assesses smallholder farmers’ adaptation options to climate change or variability for achieving food security at varied rate of impact) ( Bergene, 2024a). • Output of qualitative data analysis in line with climate change and extremes (climate change and extremes affecting the smallholders’ farming) ( Bergene, 2024b) Data are available under the terms of the Creative Commons Attribution 4.0 International license (CC-BY 4.0).
