# Supplementary material for: The Role of Climate Change Adaptation in Enhancing Household Food Security: A Case Study of the Hamassa Watershed Agroecologies, Southern Ethiopia
Source: F1000Res. 2025 Feb 11;14:188. [Version 1] doi: 10.12688/f1000research.160204.1 (PMC11926525; doi:10.12688/f1000research.160204.1)
Supplement: Peer review history

# Reviewer report: Reviewer response for version 1

**DOI:** 10.5256/f1000research.176057.r365885

**Published:** 2025-03-20

**Authors:** Souryabrata Mohapatra

**Affiliations:** 1 The University of Auckland, Auckland, Auckland, New Zealand

The study assesses the impact of climate change adaptation strategies (ACAC) on household food security in Hamassa Watershed, Ethiopia, using survey data from 328 farmers across three agroecological zones. Results show that adopting more ACAC improves food security, but effectiveness varies by region, with midlands benefiting less. Key factors influencing food security include ox ownership, crop income, and water access. The study calls for tailored climate adaptation policies to enhance food security in different agroecologies.

 My review includes feedback on methodology, analysis, and clarity, with recommendations for improvement.

**Abstract**

- Please clarify the meaning of "ACAC" before its first mention, as it is not immediately clear to readers.
- Please specify what "percent rate of 12.4, 16.3,18, and 27.7" refers to—whether it is a percentage increase in food security, household income, or another metric.
- Please rephrase "the same meaning was obtained from other food security measuring tools" to explicitly state what this means in relation to the results.
- Please explain why midlands have a negative likelihood for climate adaptation compared to highlands while lowlands have positive but insignificant implications.
- Please refine the concluding sentence to highlight how policy and technical interventions should be tailored for different agroecologies.

**Introduction**

- Please ensure proper citations for statements such as "Though the continent Africa contributes less to anthropogenic climate change, it receives the highest/damaging stress in the agriculture sector."
- Please define key terms like "climate change adaptation choices (ACAC)" earlier in the introduction.
- Please provide specific examples of successful adaptation strategies from existing studies to establish context for your research.
- Please discuss the theoretical framework in more depth—how does this study fit within broader climate change adaptation theories?
- Please elaborate on how agroecology specifically influences adaptation strategies beyond general climatic effects.
- Please clarify what "standard measure" of food security refers to in "connecting agroecology, climate change adaptation, and food security with its standard measure."
- Please justify why the study focuses on Hamassa watershed—what makes it a representative or unique study area for Ethiopia?

**Methods**

**Study Area**

- Please specify the rationale for selecting Hamassa watershed beyond geographic convenience.
- Please ensure consistent formatting for latitude and longitude coordinates, as there is some inconsistency in notation.
- Please provide more details on how agroecological classifications were determined—are they based on traditional classification, government zoning, or empirical data?
- Please include more recent references for climate data, as some cited sources are over a decade old.

**Farming**

- Please clarify how the study accounts for interannual rainfall variability when assessing food security impacts.
- Please elaborate on the specific crops grown in each agroecological zone and their vulnerability to climate variability.
- Please define the terms 'Kiremt' and 'Belg' explicitly for readers unfamiliar with Ethiopian rainfall seasons.

**Population**

- Please discuss how high population density affects adaptation strategies—does it lead to land fragmentation, overuse, or different cropping systems?
- Please provide comparative statistics with other regions in Ethiopia to contextualize Wolaita Zone’s population density.

**Sampling**

- Please justify why 328 households were selected when the Cochran formula initially suggested 384.
- Please provide more details on how households were selected proportionally from each agroecology zone.
- Please ensure the methodology accounts for gender representation and how female-headed households might have different adaptation strategies.
- Please clarify if focus group discussions (FGDs) and key informant interviews (KIIs) were transcribed and how they were analyzed.

**Food Security**

1. Please clarify why these three specific food security proxies (FBM, HDDS, CSI) were chosen over others such as food expenditure ratios or dietary energy deficiency.
2. Please define how kilocalorie calculations were standardized per household, particularly for varying household sizes.
3. Please explain why the study used a seven-day recall period for HDDS instead of a 24-hour recall, which is common in similar studies.
4. Please specify how the CSI weighting was determined for different coping strategies.

**Framework**

- Please elaborate on why multinomial endogenous switching regression was chosen over alternative models like propensity score matching or difference-in-differences.
- Please ensure clear differentiation between the multinomial logit selection model and the endogenous switching regression model—how do they complement each other?
- Please provide justification for the instrumental variables used in the 2SLS regression—why were climate perception and development agents’ support selected?
- Please clarify if independent t-tests were conducted separately for each agroecological zone or for the full sample.
- Please specify any tests conducted to check for multicollinearity in the regression models.

**Results**

- Please provide a clearer explanation of what "negative likelihood propensity" means in the context of adaptation choices.
- Please explicitly define what "return of highest utility" means when discussing ACAC adoption decisions.
- Please interpret the significance of a 12.4% increase in kcal—does this translate to food sufficiency for a certain proportion of households?
- Please explain why coping strategy differences between adopters and non-adopters are not statistically significant in some cases.
- Please ensure that all tables include units of measurement for clarity.
- Please discuss how adoption choices differ among male- and female-headed households.

**Discussion**

- Please clarify why adaptation choices beyond four strategies show diminishing or negative returns in some cases.
- Please discuss how financial constraints influence adaptation levels—are households with higher assets more likely to adopt multiple ACAC?
- Please provide a deeper analysis of the observed differences between highland, midland, and lowland adaptation outcomes.
- Please discuss potential biases in self-reported adaptation measures—how reliable is household recall data?
- Please explain the policy implications of the findings in greater detail—what specific interventions would be most effective in the midlands?
- Please incorporate more discussion on maladaptation risks, especially for households that may be adopting ineffective or counterproductive strategies.
- Please compare the findings with similar studies conducted in Ethiopia or other sub-Saharan African regions.
- Please consider discussing potential climate projections and how they may influence future adaptation strategies.

**Conclusions**

- Please refine the recommendations to specify which institutions (e.g., government agencies, NGOs) should implement them.
- Please ensure recommendations are clearly linked to empirical findings—e.g., how exactly should land rental markets be reformed?
- Please address any limitations in the study, such as potential biases in self-reported data or sample representativeness.
- Please explicitly state if follow-up studies or longitudinal research would be needed to track long-term adaptation outcomes.
- Please clarify whether policy recommendations should differ for different agroecological zones.

**Ethical**

- Please provide additional information on how oral consent was documented and whether any ethical concerns were raised by participants.
- Please explain how confidentiality of participants’ responses was ensured, especially for sensitive topics like food insecurity.

**Others**

- Please update citations with more recent studies where available, especially regarding climate change projections.
- Please check for consistency in referencing format, particularly for journal articles and government reports.
- Please ensure that all sources are properly cited within the text—some statements currently lack citations.
- Please improve figure readability by ensuring clear labels and legends, particularly for maps.
- Please provide additional context in figure captions to explain what each visualization illustrates.

Is the work clearly and accurately presented and does it cite the current literature?

Yes

If applicable, is the statistical analysis and its interpretation appropriate?

Yes

Are all the source data underlying the results available to ensure full reproducibility?

Partly

Is the study design appropriate and is the work technically sound?

Partly

Are the conclusions drawn adequately supported by the results?

Yes

Are sufficient details of methods and analysis provided to allow replication by others?

No

Reviewer Expertise:

environmental economics, agricultural economics

I confirm that I have read this submission and believe that I have an appropriate level of expertise to confirm that it is of an acceptable scientific standard, however I have significant reservations, as outlined above.

# Reviewer report: Reviewer response for version 1

**DOI:** 10.5256/f1000research.176057.r367414

**Published:** 2025-03-18

**Authors:** Mridusmita Debnath

**Affiliations:** 1 Indian Institute of Technology Guwahati, ICAR-Research Complex for Eastern Region Patna, Guwahati, India

The article is well hypothesized and partly well written as in few places including abstract the abbreviated form was not described in first instance, for example ACAC.

 The methodology section needs more explanation for reproducibility for a climate change expert as it involves lots of statistics such as co-efficient, t-value.

 For, the citing of current literature, in the hypothesis section more recent papers could be cited.

 For example "Assement of climate change impact on irrigation water demand for food security in rice fallow cropland areas using an integrated modeling techniq"

 Major comments:

 The title of the manuscript includes climate change, however no mention about IPCC reports were done in this article. The authors could have used or mentioned about several climate model including global and regional available, in this article.

 Minor comments:

 The article should be spell-checked as mistakes were there for example "tread-off".

Is the work clearly and accurately presented and does it cite the current literature?

Partly

If applicable, is the statistical analysis and its interpretation appropriate?

I cannot comment. A qualified statistician is required.

Are all the source data underlying the results available to ensure full reproducibility?

No source data required

Is the study design appropriate and is the work technically sound?

Yes

Are the conclusions drawn adequately supported by the results?

Yes

Are sufficient details of methods and analysis provided to allow replication by others?

Partly

Reviewer Expertise:

The climate change impact on agricultural water management

I confirm that I have read this submission and believe that I have an appropriate level of expertise to confirm that it is of an acceptable scientific standard.

## References

[1] : Assessment of climate change impact on irrigation water demand for food security in rice-fallow cropland areas using an integrated modeling technique. *Theoretical and Applied Climatology* .2025;156(2) : 10.1007/s00704-024-05246-5 10.1007/s00704-024-05246-5

# Reviewer report: Reviewer response for version 1

**DOI:** 10.5256/f1000research.176057.r367409

**Published:** 2025-03-04

**Authors:** Kafilah Lola Gold

**Affiliations:** 1 SARCHI-ID, University of Johannesburg, JOHANNESBURG, Gauteng, Nigeria

**Introduction**:

 The sentence where the authors claimed that no existing work may not be too correct. There are other recent and relevant work the authors either failed to look at or overlooked.

 Kindly see below:

 1. https://doi.org/10.36956/rwae.v5i4.1186 (Refer 1)

 2. https://link.springer.com/article/10.1007/s00267-023-01816-x (Refer 2)

 Also, the authors should include at the end of the introductory section the specific findings in a sentence, the recommendation, and the structure of the manuscript.

**Methods**:

 The location and physical characteristics need a more recent citation.

 The authors should use the sample determination table by *Krejcie & Morgan (1970*) to determine the accurate sample size.

 Take the conceptual framework to the beginning of the methods section. 

 The analytical framework should be changed to research design.

 The authors should include, either in the research design or in the introduction section, the theoretical justification for their study, as this is missing.

**Discussion**:

 Authors need to avoid "sexy words" sincerely. Rather, make use of more language of the trade. Link your findings to theories.

**Conclusion:**

 Also, state clearly from what period to what period the data or study was carried out.

 Recommendation, limitation, and future research direction are missing from this section, and they should be included.

**References**:

 All in-text citations must be included.

Is the work clearly and accurately presented and does it cite the current literature?

Partly

If applicable, is the statistical analysis and its interpretation appropriate?

I cannot comment. A qualified statistician is required.

Are all the source data underlying the results available to ensure full reproducibility?

Yes

Is the study design appropriate and is the work technically sound?

Partly

Are the conclusions drawn adequately supported by the results?

Partly

Are sufficient details of methods and analysis provided to allow replication by others?

Yes

Reviewer Expertise:

The necessary revisions that need to be attended to by the authors are included.

I confirm that I have read this submission and believe that I have an appropriate level of expertise to confirm that it is of an acceptable scientific standard, however I have significant reservations, as outlined above.

## References

[1] : Food Insecurity in West Africa: Is Global Warming the Driver?. *Research on World Agricultural Economy* .2024; 10.36956/rwae.v5i4.1186 403-419 10.36956/rwae.v5i4.1186

[2] : Agroecology Can Promote Climate Change Adaptation Outcomes Without Compromising Yield In Smallholder Systems. *Environ Manage* .2023;72(2) : 10.1007/s00267-023-01816-x 333-342 10.1007/s00267-023-01816-x 37004534 PMC10287806
